# The influence of catch trials on the consolidation of motor memory in force field adaptation tasks

**DOI:** 10.3389/fpsyg.2013.00479

**Published:** 2013-07-25

**Authors:** Anne Focke, Christian Stockinger, Christina Diepold, Marco Taubert, Thorsten Stein

**Affiliations:** ^1^YIG “Computational Motor Control and Learning”, BioMotion Center, Institute of Sports and Sports Science, Karlsruhe Institute of TechnologyKarlsruhe, Germany; ^2^Department of Neurology, Max Planck Institute for Human Cognitive and Brain Sciences LeipzigLeipzig, Germany

**Keywords:** motor learning, manipulandum, reaching movements, interference, after-effects, complex task

## Abstract

In computational neuroscience it is generally accepted that human motor memory contains neural representations of the physics of the musculoskeletal system and the objects in the environment. These representations are called “internal models”. Force field studies, in which subjects have to adapt to dynamic perturbations induced by a robotic manipulandum, are an established tool to analyze the characteristics of such internal models. The aim of the current study was to investigate whether catch trials during force field learning could influence the consolidation of motor memory in more complex tasks. Thereby, the force field was more than double the force field of previous studies (35 N·s/m). Moreover, the arm of the subjects was not supported. A total of 46 subjects participated in this study and performed center-out movements at a robotic manipulandum in two different force fields. Two control groups learned force field A on day 1 and were retested in the same force field on day 3 (AA). Two test groups additionally learned an interfering force field B (= −A) on day 2 (ABA). The difference between the two test and control groups, respectively, was the absence (0%) or presence (19%) of catch trials, in which the force field was turned-off suddenly. The results showed consolidation of force field A on day 3 for both control groups. Test groups showed no consolidation of force field A (19% catch trials) and even poorer performance on day 3 (0% catch trials). In conclusion, it can be stated that catch trials seem to have a positive effect on the performance on day 3 but do not trigger a consolidation process as shown in previous studies that used a lower force field viscosity with supported arm. These findings indicate that the results of previous studies in which less complex tasks were analyzed, cannot be fully transferred to more complex tasks. Moreover, the effects of catch trials in these situations are insufficiently understood and further research is needed.

## Introduction

Humans are able to learn a variety of motor skills. During practice, a neural representation of the task is formed that contains information about the physics of the musculoskeletal system and the objects in the environment. Such representations are called “internal models” (e.g., Kawato, 1999). Based on the work of Shadmehr and Mussa-Ivaldi (1994), force field studies in which subjects have to adapt to a dynamic perturbation induced by a robotic manipulandum became an established tool to study the characteristics of internal models (e.g., Malfait et al., 2002; Criscimagna-Hemminger and Shadmehr, 2008). Following acquisition, internal models undergo changes over time. This time-dependent change denotes the consolidation of a motor memory (Robertson et al., 2004; Krakauer and Shadmehr, 2006). Consolidation falls under the concept of “memory” which consists of a “bridging mechanism that retains the condensed past and present information for future use” (Tetzlaff et al., 2012). Thereby a memory can be divided in three categories: working memory, short-term memory (fragile) and long-term memory (stable) (Tetzlaff et al., 2012). Motor memory consolidation is defined as a transformation “that progresses over time from a fragile state, which is susceptible to interference by a lesion or a conflicting motor task, to a stabilized state, which is resistant to such interference” (Krakauer and Shadmehr, 2006). In contrast, learning “consists of an extraction mechanism that condenses information from past experiences and performs refinement processing of the condensed information so that behaviorally useful predictions of the future can be made” (Tetzlaff et al., 2012). Thus, motor memory consolidation denotes a specific memory process (i.e., encoding, consolidation, and retrieval) that seems to be implemented by adaptive changes in the central nervous system (Shadmehr and Holcomb, 1997; Müllbacher et al., 2001), while motor learning is a latent behavioral variable that must be inferred by changes in performance over extensive periods of time (Schmidt and Lee, 2011; Kantak and Winstein, 2012).

Evidence for consolidation of motor memories was first presented by Shadmehr and co-workers (Brashers-Krug et al., 1996; Shadmehr and Brashers-Krug, 1997). In these experiments, subjects were instructed to control a robotic manipulandum and to perform point-to-point reaching movements to predefined target locations. The robotic manipulandum is able to generate forces during arm movements (dynamic perturbations). When no forces were active (null field), subjects produced straight hand trajectories (Shadmehr and Brashers-Krug, 1997). If a clockwise velocity-dependent force field (force field A) was activated, subjects produced distorted trajectories with a characteristic “hooking” pattern. With practice, the hooks diminished and the hand trajectories in force field A became similar to those observed in the null field (adaptation). When retested after 24 h, subjects showed retention of performance in force field A indicating consolidation of the motor memory. In contrast, if subjects learned a second opposing force field B (= −A) at different time intervals after force field A, retest performance in force field A is sometimes diminished because force field B interferes with the consolidation of force field A. If the time interval between learning of field A and B was less than 5 h consolidation of force field A was absent (Shadmehr and Brashers-Krug, 1997). Only with time intervals more than 5 h a consolidation of force field A could be observed.

Impaired retest performance in force field A for the groups with less than 5 h time interval between learning force field A and B indicates that task A was affected by either retrograde or anterograde interference. Retrograde interference would be a disruption of the memory of task A from learning a new task B and is directed backward from learning B (day 2) onto learning A (day 1). In contrast, anterograde interference is directed forward and describes disruption of retest performance in force field A (day 3) due to performance in task B (day 2). Given the fact that in the above described studies interference decreased if the time between force field A and B increased, it seems likely that the interference is mostly of retrograde source (Robertson et al., 2004).

Caithness et al. (2004) failed to confirm the results of these previous studies and showed consolidation only for control groups which did not perform in force field B between test and retest in force field A (AA). Neither the test group with a 5 min nor the test group with a 24 h time interval between force field A and B (ABA) showed consolidation. Although the time interval between learning the two force fields was more than the critical 5 h, it can be assumed that the interference for the 24 h group is still of retrograde source as the time interval between learning B and retesting A was 1 week making anterograde interference improbable.

The conflicting results of Caithness et al. (2004) and Shadmehr and co-workers (Brashers-Krug et al., 1996; Shadmehr and Brashers-Krug, 1997) could be based on randomly presented catch trials, during which no forces were applied. While consolidation was found for studies including catch trails (Brashers-Krug et al., 1996; Shadmehr and Brashers-Krug, 1997), no consolidation was observed without using catch trials (Caithness et al., 2004). Overduin et al. (2006) confirmed these findings and showed that learning with catch trials leads to retention and consolidation of the initial task when two opposing force fields where learned 6 h apart, while learning without catch trials leads to interference and therefore no consolidation. They explained their results based on the assumption that random practice degrades performance in acquisition but facilitates the performance in the retention test (Schmidt and Lee, 2011). In literature, different hypotheses on these contextual interference effects are discussed. Two common theoretical explanations are the elaborative-processing hypothesis and the forgetting-reconstruction hypothesis. The elaborative-processing hypothesis suggests that random practice promotes more comparative and contrastive analyses of the actions required to learn the task. Thus, the learner can encode critical task-related information which leads to a stronger and more elaborate memory representation (Schmidt and Lee, 2011; Kantak and Winstein, 2012). The forgetting-reconstruction hypothesis is based on the assumption that the action planning prior to a trial is influenced by what has been done in the previous trial. Thereby, random practice forces the learner to forget the previously constructed action plan because the subsequent trial is different, and to reconstruct the plan again when the initial task is presented again later. This added cognitive processing might be responsible for the poorer performance during adaptation but the stronger motor memory at retention (Schmidt and Lee, 2011; Kantak and Winstein, 2012). From a more computational view the benefit of variable randomized learning is based on the assumption that motor learning is an iterative control process driven by motor error. This motor error arises by comparing the predicted sensory state provided by an (forward) internal model with the actual sensory feedback of a movement. Based on this motor error, the control mechanism is changed on the subsequent trial (Cisek, 2005; Shadmehr et al., 2010). Thus, random practice or more specifically randomized catch trials within the acquisition might enlarge the motor error and thereby improve the control mechanisms as adaptation is thought to be an iterative process that minimizes movement error from trial to trial. Several state-space models of motor adaptation act on the assumption that the prediction error on one trial is correlated to the change in motor output in the next trial (Thoroughman and Shadmehr, 2000; Smith et al., 2006; Lee and Schweighofer, 2009). Such models can account for most phenomena of motor learning as savings, anterograde, and retrograde interference as well as spontaneous recovery. This strengthens the assumption that the motor error plays a critical role in the adaptation and consolidation process.

Other authors also referred to the results of Overduin et al. (2006) and assigned catch trials a significant role in the process of consolidation (Criscimagna-Hemminger and Shadmehr, 2008). However, we found only one study with a very specific measurement setup (Overduin et al., 2006) that could confirm the theory that unpredictable interruptions in force field learning result in increased stabilization of motor memory (Overduin et al., 2006). The viscosity of the force field in previous studies that found consolidation with catch trials was set at 10–13 N·s/m (Brashers-Krug et al., 1996; Shadmehr and Brashers-Krug, 1997; Overduin et al., 2006) and the upper-arm of the subjects was supported in the horizontal plane by a rope attached to the ceiling while performing the point-to-point reaching movements. Thereby, the redundant degrees of freedom (Bernstein, 1967) of the subjects' arms were partly constraint simplifying the movement task to be learned. In natural reaching movements the humans' arms are free to move denoting a higher number of degrees of freedom to be coordinated during task execution. In addition, in the above cited studies dynamic environments were simulated in which the effective endpoint mass was about 0.26 kg. However, one of the defining characteristics of human life is the ability to use different tools with diverse dynamic properties (Criscimagna-Hemminger and Shadmehr, 2008). To the best of our knowledge, the combination of unconstrained arm movements with higher simulated endpoint masses have not yet been studied in the context of consolidation experiments. We are of the opinion that it is essential to unravel whether the current findings (Brashers-Krug et al., 1996; Shadmehr and Brashers-Krug, 1997; Overduin et al., 2006) are also valid for more complex movement tasks and therefore can be generalized.

Thus, the aim of this study was to investigate whether the presence or absence of catch trials during force field adaptation also influences consolidation in more complex tasks. In order to relate the present findings to previous force field setups, we used force fields of higher viscosity as well as an unsupported arm configuration during force field adaptation. We hypothesized that, despite a higher viscosity of the force field and an unsupported arm, catch trials lead to consolidation of motor memory when learning two opposing force fields one day apart.

## Materials and methods

### Subjects

A total of forty-six adults (age: 24.1 ± 2.6 yrs, male: 30, female: 16) participated in this study. They all gave written informed consent and the test-protocol was reviewed and approved by the Institutional Review Board. All subjects were naive to the experimental procedure and had no obvious motor deficits. The 46 participants were randomly assigned to four groups of 11–13 subjects each, whereas two control groups (C1, C2) and two test groups (T1, T2) were defined. All subjects learned to make reaching movements while interacting with a force produced by a robotic manipulandum. While the control groups learned force field A and were retested under the same force field condition A without an interfering task between learning and retest (AA), the test groups had to learn a second interfering force field B between learning and retest of force field A (ABA). The difference of group 1 and 2 was defined by the absence (0%) or presence (~19%) of catch trials, respectively.

### Apparatus

**Figure 1:**
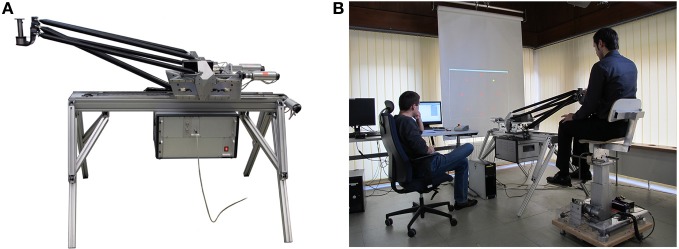
**Robotic manipulandum “BioMotionBot” (A), setting of the experiment (B)**.

Subjects held the handle of a robotic manipulandum (“BioMotionBot”, Figure 1A) that could exert forces (Bartenbach et al., 2013). The arm of the participants was not supported and all movements were restricted to the horizontal plane. Subjects were seated on a chair that was locked into an individual position enabling the subjects to grasp the handle with their dominant hand (Figure 1B). Visual feedback of hand position and targets was provided on a screen mounted above the manipulandum (Figure 1B).

### Procedure

#### Task

**Figure 2:**
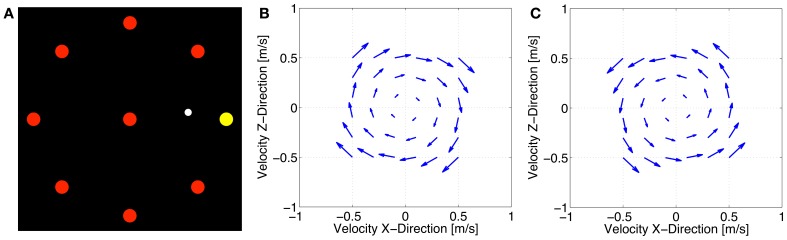
**Center-out task (A), velocity-dependent clockwise force field (B), and velocity-dependent counterclockwise force field (C)**.

Subjects used the handle of the manipulandum to make visually guided reaching movements. During these movements, the arm was not supported. Subjects had to perform center-out tasks to eight targets around the center (Figure 2A). Each target was visualized as a 1 cm wide red circle on a black background while the cursor had a diameter of 0.3 cm. Distance from the center to each of the eight targets was 10 cm in real space. Starting from the center subjects had to reach for each of the eight circumjacent targets in pseudo-randomized order and then move back to the center. When the current target was reached, the next target flashed up. Targets that should be reached next changed its color from red to yellow. One block was defined by 16 trials (eight inwards and outwards movements, respectively) and each of the eight targets was presented once per block. Participants were given 500 ± 50 ms to complete each movement, starting from the moment the current target was left and lasting until the cursor entered the next target. Reaction time was excluded from this time interval. If the subjects reached the target within the allotted time, a green circle around the target appeared. If they moved too slowly, a red circle appeared and when moving too fast an orange circle turned up. This visual feedback was provided throughout the entire experiment.

#### Design

On day 1, all subjects performed a familiarization block under null field condition (no forces) for 25 blocks (400 trials) in which all eight targets were presented. Generally, after these 400 trials subjects were able to move at the required pace. No further analysis was made of this data. After a 5 min rest, subjects performed again six blocks (96 trials) in a null field. Based on these trials, so-called baseline trajectories were calculated. These are commonly used as reference to movements conducted under force field conditions. After another 5 min rest, subjects completed a learning block of 16 blocks (256 movements, according to Caithness et al., 2004 and Overduin et al., 2006) in a velocity-dependent clockwise force field A. On day 2, the test groups performed an interference block of 16 blocks (256 trials) in a velocity-dependent counterclockwise force field B. All groups returned on day 3 and experienced the same force field A of day 1 in a retest block (Table 1). Control and test group 1 (C1, T1) performed all 256 trials in the force field while the catch trial groups (C2, T2) performed only 208 trials in the force field and 48 (~19%) catch trials without forces (null field). Visual feedback was only provided to help the subjects making their movements in the required time but not to evaluate any success. After the baseline block, subjects were told that they now would experience “forces” generated by the robotic manipulandum. They also were instructed to sleep at least 6 h between the sessions.

**Table 1:** **Description of the experiment**.

**Group**	**Subjects**	**Catch trials**	**Paradigm**		
			**Day 1**	**Day 2**	**Day 3**
Control 1	*n* = 13	No	F N A		A
Control 2	*n* = 11	Yes (19%)	F N A		A
Test 1	*n* = 11	No	F N A	B	A
Test 2	*n* = 11	Yes (19%)	F N A	B	A

*F, familiarization in null field; N, baseline in null field; A, velocity-dependent clockwise field; B, velocity-dependent counterclockwise field*.

#### Forces

All subjects started the experiment with a familiarization block to learn to move at the required pace followed by a baseline block. In both cases, all subjects experienced a null field. Afterwards, two velocity-dependent force fields were generated by the BioMotionBot that were proportional to the reaching speed and always pushed the handle perpendicular to its current movement direction in a clockwise (A) or counterclockwise (B) direction (Figures 2B,C). It was generated using the following equation:

$$\left[\begin{array}{l} Fx\\ Fz \end{array}\right]=k\left[\begin{array}{cc} \text{cos}(\theta ) & -\sin(\theta )\\ \sin(\theta ) & \text{cos}(\theta ) \end{array}\right]\left[\begin{array}{l} \dot{x}\\ \dot{z} \end{array}\right]$$

where *Fx* and *Fz* are robot-generated forces, *k* = 35 N·s/m indicates the force field viscosity, θ = −90° (clockwise) or 90° (counterclockwise), $\dot{x}$ and $\dot{z}$ are the components of the hand velocities in the horizontal plane.

#### Catch trials

While control and test group 1 performed all trials under force field conditions, for the control and test group 2, 48 catch trials (~19%, according to Shadmehr and Brashers-Krug, 1997 and Overduin et al., 2006) were embedded within the 16 blocks. On these trials the force field was turned off suddenly without announcement. Catch trials were programmed pseudo-randomly and occurred on the same trials for both catch trial groups (C2, T2) and both force fields (A, B). The trajectories that result during catch trials when the robot is producing a null field unexpectedly are called “after-effects”.

### Analysis

All parameters were calculated using the custom made software “ManipAnalysis” (Stockinger et al., 2012). Therewith, raw data of the hand trajectories were filtered using a fourth-order Butterworth low-pass filter with a cut-off frequency of 10 Hz. Based on the filtered data movement velocities were calculated. All time derivatives were numerically computed by central difference method. Afterwards, the data sets were segmented. Our segmentation algorithm is based on the idea that the movement starts if the cursor leaves the starting point and ends if the cursor reaches the target point. In the last preprocessing step, the segmented data sets were time normalized in order to make them comparable. Time normalization was executed via cubic spline interpolation of recorded data points and subsequent rescaling the sampling rate as a percentage of the duration (Stockinger et al., 2012).

To quantify performance, for each trial in the force field the enclosed area between the hand trajectory and the straight line joining start and target point was calculated according to Caithness et al. (2004). To analyze after-effects indicated by the performance of the catch trials we used a directed distance measure according to Shadmehr and Brashers-Krug (1997) that quantified the maximum perpendicular distance of the hand path from a straight line to the target. The direction was positive for clockwise deviations (after-effect appropriate to force field B) and negative for counterclockwise deviations (after-effect appropriate to force field A).

### Statistics

Mean scores of the first and last block were calculated and compared, whereat catch trials were excluded from the calculation of these mean scores (Caithness et al., 2004). Single factor ANOVAs were assessed to compare the initial as well as the end performance in force field A on day 1 between the four groups, respectively. *T*-Tests were conducted to analyze differences between the initial and the end performance for each group separately.

The consolidation of force field A was estimated by comparing the initial performance on the learning session on day 1 and the retest session on day 3. Main and interaction effects of the within-subject-factor time (initial performance on day 1 and day 3) and the between-subject-factor group (with or without catch trials) were assessed for the control and test groups separately using repeated-measures ANOVAs (2 × 2). The level of significance for all tests was set a priori to 0.05.

## Results

To analyze the effect of catch trials on the consolidation of motor memory, subjects learned to make reaching movements at a robotic manipulandum in two different dynamic environments (force field A and B) with and without catch trials, respectively. The task was more complex than in previous studies as the viscosity of the force fields was increased and the upper arm was not supported during the movements. We tested for the stability of the adapted field A as a function of presence or absence of catch trials.

### Adaptation to a novel dynamic environment

**Figure 3:**
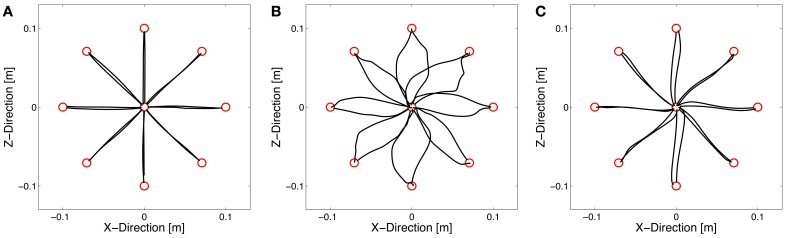
**Hand trajectories of one subject (group T1) under null field condition (A), at the beginning of learning force field A (B), and at the end of learning force field A (C)**. The subject showed straight trajectories in the null field, highly distorted trajectories at the beginning of force field training, and again almost straight hand trajectories at the end.

**Figure 4:**
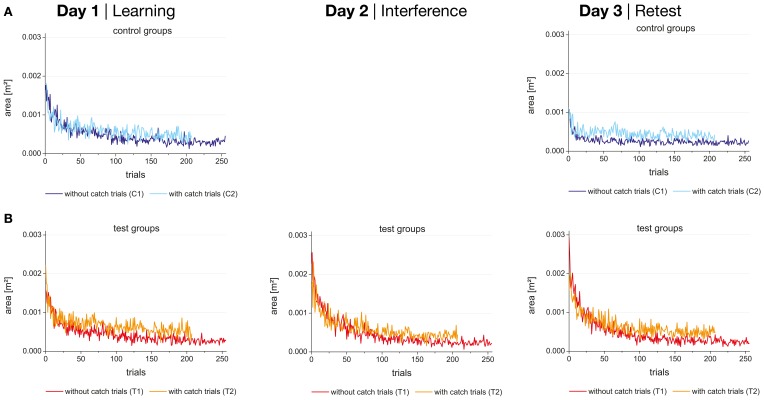
**Performance (enclosed area) during the 256 trials (without catch trials) or 208 trials (with catch trials) in force field A on day 1 (learning), force field B on day 2 (interference), and force field A on day 3 (retest) for control (A) and test groups (B) separately**.

Subjects started to make reaching movements in the null field without disturbing forces. A typical hand trajectory of one subject is shown in Figure 3A. The movements are approximately in a straight line. After introducing a velocity-dependent force field, the hand trajectories became highly distorted (Figure 3B). The force field pushed the hand of the subjects in a direction perpendicular to the movement direction resulting in a characteristic “hooking” pattern. However, with practice the hand trajectories converged toward a straight line (Figure 3C), typically produced by subjects under null field conditions (Flash and Hogan, 1985). Accordingly, the enclosed area decreased with practice (Figure 4).

Figure 4A shows the performance (enclosed area) during learning block (day 1) and retest block (day 3) of force field A for the control groups (with and without catch trials) which experienced no interfering force field on day 2. The performance of the test groups which experienced an interfering force field B on day 2 is shown in Figure 4B. As expected the enclosed area decreased with practice for all four groups.

No significant differences in the initial performance of force field A on day 1 (mean score of the first block) were found between the four groups. Additionally, performance was for all four groups significantly different between the first block (mean score) and the last block (mean score) indicating that all four groups were able to adapt to force field A within 256 trials (*p* < 0.001 for all four groups). Comparing the end performance of force field A on day 1 between the four groups the test group without catch trials (T1) showed significantly lower values than the test group with catch trials (T2) (*p* < 0.001) indicating a putative better adaptation to the force field for the test group without catch trials. However, a repeated-measures ANOVA (2 × 2) showed no significant interaction between group and time when comparing the initial and end performance of day 1 for the two test groups. This detects a similar adaptation of force field A on day 1 for both test groups. For the control groups, no significant difference between the values at the end of force field A was revealed and comparing the corresponding two catch trial and no catch trial groups significant differences were neither found between C1 and T1 nor between C2 and T2. Thus, we act on the assumption that the base level of the four groups after day 1 was similar.

The progressions of the catch trial performance indicating the after-effects are shown in Figure 6 for both catch trial groups (C2, T2). Catch trial performance developed in the opposite direction than the force field trial performance starting with a perturbation close to zero increasing during the 48 catch trials and showing a performance plateau after the first 20 catch trials. Thereby, the direction of the deviation depends on the force field that was learned (clockwise or counterclockwise). While the force field trial performance improved during dynamic learning the catch trial performance worsened during the adaptation favoring the idea that improvement in force field trial performance was due to formation of an internal model rather than a simple increase in stiffness of the arm (Shadmehr and Mussa-Ivaldi, 1994).

### Consolidation

To investigate the influence of catch trials on the consolidation we compared the initial performance in field A of day 1 and day 3 for the two control groups and the two test groups, respectively, including time as within-subject-factor (initial performance on day 1 and day 3) and group as between-subject-factor (with or without catch trials).

**Figure 5:**
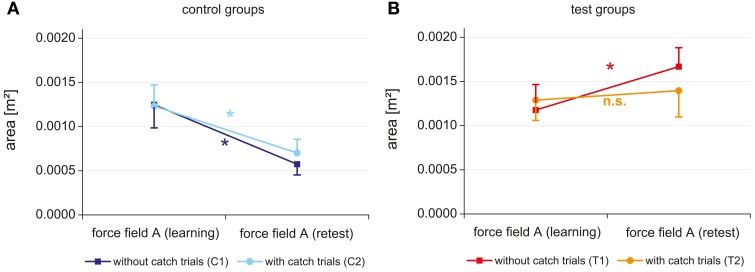
**Initial performance (mean score of the first block) in force field A of day 1 and day 3 for the control groups with and without catch trials (A) and the test groups with and without catch trials (B)**.

For the control groups a significant time effect (*p* < 0.001, η^2^ = 0.874) was revealed (Figure 5A). No significant group effect and no significant interaction between time and group were found, indicating a comparable improvement of performance from day 1 to day 3 for both control groups (with and without catch trials).

In contrast, different results were revealed for the test groups, which experienced an interfering force field B between learning and retest of field A. Besides a significant time effect (*p* < 0.001, η^2^ = 0.530) and no significant group effect the ANOVA showed a significant interaction between time and group (*p* = 0.006, η^2^ = 0.316). While the test group T2 with catch trials did not change its initial performance in force field A from day 1 to day 3, the test group T1 without catch trials even degraded the initial performance in field A from day 1 to day 3 (Figure 5B). Thus, interference effects were shown for both test groups and further analysis must expose if these interferences are of retrograde or anterograde source.

### Retrograde and anterograde interference

As we found no consolidation of force field A for both test groups, either retrograde interference, anterograde interference, or both types of interference in combination must be detectable. To test for retrograde interference from task B (day 2) onto learning of task A (day 1), previous studies altered the temporal distance between learning force field A and force field B. By analyzing after-effects of the catch trials they showed that up to a temporal distance of 5.5 h learning of B starts in an internal model appropriate for A which might result in an unlearning of A (Shadmehr and Brashers-Krug, 1997).

**Figure 6:**
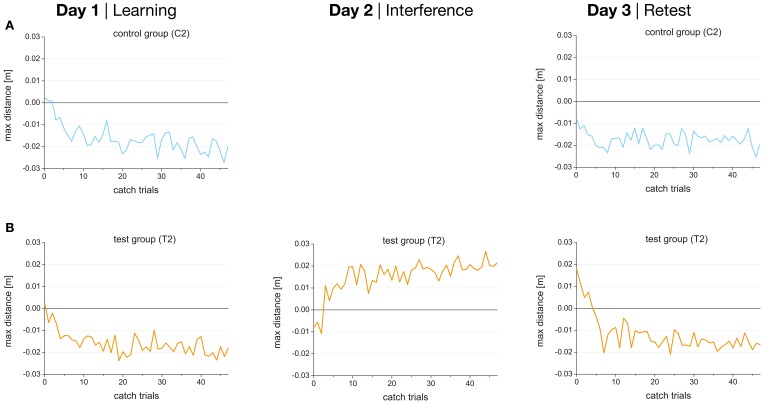
**Performance (maximum perpendicular distance) during 48 catch trials in force field A on day 1 (learning), force field B on day 2, and force field A on day 3 (retest) for control (A) and test groups (B) separately**. Negative values indicate after-effects appropriate to force field A, and positive values indicate after-effects appropriate to force field B.

The results of the present study showed that learning of B for the test group with catch trials (T2) started in an internal model appropriate for A (indicated by the negative values for the first catch trials in force field B; Figure 6B, mid). This might lead to an unlearning of task A and would therefore be interpreted as retrograde interference. However, as we did not change the time interval between learning A and B we cannot be sure that retrograde interference influenced the consolidation process of A. In contrast, anterograde interference can be determined by analyzing the after-effects of retest A on day 3. Data showed that the after-effects in force field A on day 3 started in direction of force field B (Figure 6B, right) indicating anterograde interference from force field B (day 2) onto retest of force field A (day 3). This anterograde interference could also have disrupted the consolidation of A.

## Discussion

The purpose of this study was to test whether the presence or absence of catch trials during force field adaptation in more complex tasks could influence the process of consolidation similarly to the study of Overduin et al. (2006). In contrast to previous experiments (Brashers-Krug et al., 1996; Shadmehr and Brashers-Krug, 1997; Overduin et al., 2006), this study was the first study in which subjects performed point-to-point reaching movements at a robotic manipulandum with an unsupported arm in a velocity-dependent force field of 35 N·s/m. Our results for the control groups showed that both groups exhibited a consolidation from day 1 to day 3 and no performance differences on day 3 between the two groups were observed. Therefore, in our study catch trials had no effect on the consolidation of motor memory in the absence of an interfering condition on day 2. In contrast to the control groups, the test groups experienced an interfering condition on day 2. The results exhibited no consolidation for group T2 (with catch trials) from day 1 to day 3 and even a decrease of performance on day 3 compared to day 1 for group T1 (without catch trials). Thus, our hypothesis that the presence of catch trials during adaptation in force fields with higher viscosity and an unsupported arm leads to consolidation of motor memory, cannot be confirmed.

Our results are partly in line but also in contrast to previous studies that investigated the influence of catch trials on the consolidation of motor memory. Similar to all previous studies (Shadmehr and Mussa-Ivaldi, 1994; Brashers-Krug et al., 1996; Shadmehr and Brashers-Krug, 1997; Caithness et al., 2004; Overduin et al., 2006), our subjects were able to adapt to different force fields as the enclosed area decreased significantly during the 256 trials in each session on day 1 and day 3. Moreover, the time courses of the adaptation profiles (Figure 4) revealed that the subjects' performance expose only fluctuations of small magnitudes during the last quarter of the training sessions indicating almost a steady state behavior. We also found retention of force field A for both control groups that experienced only one force field. These findings support the idea that an internal model can be consolidated and become available for “recall” even for a movement task in a force field with higher viscosity and with an unsupported arm. Comparable to Caithness et al. (2004) and Overduin et al. (2006), our test group without catch trials did not show consolidation of force field A. They even decreased in performance on day 3 compared to day 1. But in contrast to Brashers-Krug et al. (1996), Shadmehr and Brashers-Krug (1997), and Overduin et al. (2006) we did not find consolidation of force field A for the test group that learned with catch trials. However, catch trials had a positive effect in the ABA paradigm if we take into account that the test group that learned with catch trials (T2) showed a significant better initial performance on day 3 than the test group that learned without catch trials (T1). In combination with the fact that the test group with catch trials showed a slightly worse performance at the end of day 1 a contextual interference effect could be shown for the test groups. Therefore, catch trials positively influenced the performance of day 3 even in more complex tasks which is in line with the idea of variable learning.

Possible explanations that could account for the fact that we did not find consolidation when both force fields (A, B) were experienced with catch trials and one day apart, even though there was a tendency toward a positive catch trial effect, are considered in the following discussion: (1) the higher viscosity of the force field, (2) the unsupported arm, (3) the effect of anterograde interference, and (4) the chosen parameter and the definition of consolidation.

First, in our study the viscosity of the force field was more than double the viscosity in studies that found consolidation when learning with catch trials. This higher viscosity might have influenced the consolidation process of force field A. However, as the control groups where able to consolidate force field A despite the higher viscosity it cannot be concluded that the higher viscosity *per se* leads to a failure of consolidation. Additionally, all groups were able to adapt to the force fields within 256 trials to a performance plateau. This indicates that the viscosity was not too high to enable the subjects adjusting their performance within the given task to a sufficient level. Nevertheless, when subjects learned an interfering force field B no consolidation was found for both groups, with and without catch trials. It was just shown that the test group with catch trials performed better on day 3 than the test group without catch trials. Overduin et al. (2006) concluded based on the forgetting-reconstruction hypothesis that catch trials are unpredictable interruptions causing active unlearning and relearning of the novel condition and thus lead to a greater stabilization in memory. Our results do not entirely confirm this assumption as we also implemented pseudo-randomized catch trials in terms of unpredictable interruptions but did not find consolidation of force field A.

Second, in all previous studies that found consolidation when learning with catch trials, subjects' arms were supported in the horizontal plane by a rope attached to the ceiling while performing the point-to-point reaching movements (Brashers-Krug et al., 1996; Shadmehr and Brashers-Krug, 1997; Overduin et al., 2006). Caithness et al. (2004) tested for the effect of arm support and showed no consolidation for both conditions, with and without supported arm. However, in both experiments subjects learned without catch trials. Thus, only the combination of supported arm and catch trials seems to lead to consolidation of force field A. If the support of the arm is a critical issue this might be an explanation of our results. The reduction of the degrees of freedom of the arm leads to a restricted movement and thus to a lower complexity of the task. The complexity of the task, in turn, might play an important role in the consolidation process of motor memory. In combination with the higher viscosity of the force field the unsupported arm in the present study might have inhibited a consolidation of force field A for the test groups as the complexity of the task was too high. Maybe an increase of trial number would enhance the performance at retention test, since previous studies showed positive effects of overlearning on the consolidation process (Driskell et al., 1992; Yin and Kitazawa, 2001). Besides the amount of practice the variability of practice (Schmidt and Lee, 2011) in terms of a higher rate of catch trials might have a more positive effect on motor memory consolidation as discussed above. Although the relationship between dynamic learning and motor skill learning (e.g., learning to tie shoe laces, playing golf) is not fully understood (Huang and Krakauer, 2009; Yarrow et al., 2009), a higher rate of catch trials could trigger similar positive effects as reported in motor skill learning in connection with variable practice (Schmidt and Lee, 2011).

Third, it has also been suggested that poor initial performance on the retest of A may rather be explained by anterograde interference of B on the second experience of A than by retrograde interference (Miall et al., 2004; Overduin et al., 2006). Previous studies that analyzed consolidation processes using the ABA paradigm changed the time interval between learning A and B to check for retrograde interference (Brashers-Krug et al., 1996; Shadmehr and Brashers-Krug, 1997; Caithness et al., 2004). As they found consolidation of A only when the time interval between learning A and B was higher than 5 h they concluded that this consolidation of A was achieved due to a decrease of retrograde interference. Shadmehr and Brashers-Krug (1997) additionally analyzed after-effects of the included catch trials and found that the learning of force field B on day 2 started in an internal model appropriate to A for all groups with time intervals less than 5 h between learning A and B. Thus, they concluded that the internal model of A is used to learn B, leading to an unlearning of A and thereby to a catastrophic interference. Only for the groups with time intervals higher than 5 h learning of B started in an internal model that was close to a “tabula rasa”. However, later studies (Pekny et al., 2011) showed that training in two opposing force fields with no temporal difference between the two training episodes produces two competing memories and not destruction or overwriting of one memory. In the present study, the test group with catch trials also started learning B in an internal model appropriate to A. According to Pekny et al. (2011) who found that two opposing force fields do not destroy each other this should rather be interpreted as anterograde interference from learning A onto learning B than retrograde interference from learning B back onto learning A. This is also supported by the fact that learning of B started with significant higher perturbations than learning A for both test groups, with and without catch trials (data not shown in the results section: time effect: *p* = 0.002, η^2^ = 0.384, group effect: n.s., interaction: n.s.). If anterograde interference can be found from day 1 onto day 2 it is probable that anterograde interference can also be found from day 2 onto retest on day 3. We found that retest of A on day 3 started in an internal model appropriate to B for the test group with catch trails. This could explain the fact that no consolidation of A was observed. However, we cannot be sure that this was also the case for the test group without catch trials as we were not able to analyze after-effects for this group. In the present study, subjects had a rest of 24 h between learning B on day 2 and retesting A on day 3. Overduin et al. (2006) showed consolidation of A with a similar rest between learning B and retesting A but used a force field with lower viscosity and supported the arms of the subjects. For such simpler tasks a rest of 24 h seems to be enough to prevent anterograde interference. However, for a more complex task this period might be too short and must be extended or so called wash out trials must be embedded (Krakauer et al., 2005).

Fourth, the parameter to describe adaptation and consolidation in force field tasks varies among studies. All studies that found consolidation in the ABA paradigm used velocity vector correlation to describe the performance of the subjects (Brashers-Krug et al., 1996; Shadmehr and Brashers-Krug, 1997; Overduin et al., 2006). Velocity vector correlation quantifies the similarity between subjects' velocity profiles in the force field with corresponding velocity profiles in the baseline. Thereby, Shadmehr and co-workers selected individual baseline speed vectors that were optimally correlated to subsequent vectors while Overduin et al. (2006) calculated baseline vectors as the average of the last four baseline trials. In contrast, we used the same parameter as Caithness et al. (2004) that is based on the hand trajectories. Here, the enclosed area between the hand trajectory and the straight line joining start and target is calculated. Neither Caithness et al. (2004) nor we found consolidation of force field A with an interfering force field B using this trajectory based parameter. Moreover, the statistical calculation of consolidation is different in previous studies. While some compared the mean performance of the entire learning block (day 1, force field A) with the entire retest block (day 3, force field A) (Brashers-Krug et al., 1996; Shadmehr and Brashers-Krug, 1997) others compared the initial performance of the learning and retest block (Caithness et al., 2004). Overduin et al. (2006) compared the final performance of learning with the initial performance of retest. Thus, the combination of adaptation parameter and statistical calculation leads to three different methods to determine consolidation. Maybe, not the catch trials are responsible for the differences of previous studies but the calculation methods to determine consolidation is critical. Further studies should check on differences in the results when using different calculation methods to rule out methodical effects on the results.

To our knowledge, this is the first study that investigated consolidation of motor memory in force field studies with the ABA paradigm using a velocity-dependent force field with a high viscosity of 35 N·s/m and an unsupported arm. Such an increase of task complexity seems to lead to different consolidation patterns. Although we cannot support the results of previous studies (Brashers-Krug et al., 1996; Shadmehr and Brashers-Krug, 1997; Overduin et al., 2006), we also found different performance on day 3 for the group that learned with catch trials compared to the group without catch trials. Thus, catch trials seem to have a positive effect on the performance on day 3 even for more complex movement tasks (higher viscosity, unsupported arm). However, there might also be other factors like methodical aspects that affect the results of the present and previous studies.

Further studies should continue concentrating on tasks of different complexity as the consolidation of motor memories needs to be investigated in various settings to be understood sufficiently. For more complex motor tasks, a higher number of trials or catch trials might be necessary to find similar consolidation effects as in previous studies. The effect of methodical differences on the results should be investigated and to prevent from anterograde interference time intervals between learning B and retesting A could be prolonged or washout trials should be included at the start of each session (Caithness et al., 2004; Krakauer et al., 2005).

### Conflict of interest statement

The authors declare that the research was conducted in the absence of any commercial or financial relationships that could be construed as a potential conflict of interest.
